# Editorial: Oligomerization and fibrillation of amyloid peptides: Mechanism, toxicity and inhibition

**DOI:** 10.3389/fmolb.2022.1023047

**Published:** 2022-09-20

**Authors:** Zhenyu Qian, Nabanita Saikia, Yunxiang Sun

**Affiliations:** ^1^ Key Laboratory of Exercise and Health Sciences (Ministry of Education), School of Kinesiology, Shanghai University of Sport, Shanghai, China; ^2^ Chemistry Department, School of Science, Ivan Hilton Science Center, New Mexico Highlands University, Las Vegas, NM, United States; ^3^ Department of Physics, Ningbo University, Ningbo, China

**Keywords:** Amyloid peptide, protein aggregation, protein-nanoparticle interaction, protein-membrane interaction, inhibitory mechanism

The pathological process of neurodegenerative diseases is closely related to the aggregation of one or more types of amyloid peptides, such as β-amyloid (Aβ) and tau in Alzheimer’s disease (AD), human islet amyloid polypeptide (hIAPP) in type 2 diabetes (T2D), α-synuclein (αS) in Parkinson’s disease, etc. Accumulating evidences shows that the neurotoxicity comes from the oligomerization and fibrillation of amyloid peptides. However, the formation of amyloid fibrils is highly complex and influenced by multiple factors. To fundamentally explore the mechanism, toxicity and inhibition involved in the oligomerization and fibrillation of amyloid peptides will facilitate the detection and mitigation of neurodegenerative diseases. In this Research Topic, four original research articles have been published, dedicated to molecular structures and interactions of amyloid peptides.

The hetero-aggregates of Aβ and IAPP may be responsible for a pathological link between AD and T2D. Adem et al. presented experimental and computational characterizations of the kinetic profiles, morphologies, secondary structures and toxicities of IAPP-Aβ40 heteroassemblies, and compared them to those formed by their homoassemblies. Monomeric IAPP and Aβ40 were found to form stable heterodimers first, followed by β-sheet-rich heteroaggregates which are toxic to both neuronal and pancreatic model cells. Then, epigallocatechin gallate (EGCG) was selected to inhibit IAPP-Aβ40 co-aggregation. It was demonstrated to reduce heteroaggregate formation and β-sheet content, and effectively reduce the toxicity of IAPP-Aβ40 hetero-aggregates on both cell models when the concentration ratio to peptides is higher than 2.5-fold. The study by Adem et al. highlights the co-aggregation pathway of IAPP and Aβ40, and its inhibition by EGCG contributing to a preventative therapy against the T2D-AD association.

The peptide-lipid interactions may influence the fibrillation and toxicity of amyloid peptides. Dubackic et al. investigated the morphology of αS fibrils formed under different conditions and the influence of lipid membranes by small and wide-angle X-ray scattering. They showed that the observed fibril corresponds to a fibril structure composed of two protofilaments, and the lipid to peptide ratio (0–7.5) and pH (6.0–7.0) have an ignorable effect on the cross-section radius and β-strand repeat distance of the fibril. The study by Dubackic et al. helps to understand the effect of phospholipid concentration and pH on the structure of αS fibrils.

Post-translational modifications such as acetylation, phosphorylation, nitration, etc., may regulate the physiology of amyloid peptides. Zou and Guan investigated the effect of K280 acetylation on the aggregation of hexapeptide in tau paired helical filament (PHF6*) by replica-exchange molecular dynamics simulations. They showed that K280 acetylation strengthens the intermolecular interactions and leads to more ordered β-sheet-rich structures, which may be attributed to the role of the hydrophobic residues in PHF6* peptides. The study by Zou and Guan further deepens the understanding of the relationship between acetylation and tau aggregation.

Macrochirality of supramolecular peptide structures is vital in biological activities. Guo et al. studied the self-assembled morphologies of two chiral amyloid peptides Ac-KHHQKLVFFAK-NH_2_ (KK-11, L-amino acids) and KKd-11 (D-amino acids). Topological characterizations by atomic force microscope and transmission electron microscope revealed that KK-11 or KKd-11 peptides, respectively self-assemble into right- or left-handed helical nanofibrils, and only achiral nanowires are formed when the two peptides are mixed in a wide range of concentration ratios. Circular dichroism and Fourier transform infrared spectra indicated that the secondary structures are changed when the peptides co-assemble. The assembled nanofibrils by molecular dynamics simulations are in good agreement with experimental observation, and more interestingly, the peptides tend to co-assemble instead of self-sort. The study of Guo et al. sheds light on the molecular mechanisms of the macrochirality of supramolecular peptide structures, which facilitates the exploration of the chirality origin in biosystems and the applications of complementary-chirality designs.

In conclusion, the publications in this Research Topic have made an innovative attempt to uncover the complex mechanisms involved in molecular structures and interactions in the oligomerization and fibrillation of amyloid peptides.

